# Systematic review of school-based interventions to prevent smoking for girls

**DOI:** 10.1186/s13643-015-0082-7

**Published:** 2015-08-14

**Authors:** Miriam J.J. de Kleijn, Melissa M. Farmer, Marika Booth, Aneesa Motala, Alexandria Smith, Scott Sherman, Willem J.J. Assendelft, Paul Shekelle

**Affiliations:** 1 Gender & Women’s Health, Department of Primary and Community Care, Radboud University Medical Center, Postbus 9101, 6500 HB Nijmegen, The Netherlands; 2 VA HSR&D Center for the Study of Healthcare Innovation, Implementation, and Policy, VA Greater Los Angeles Healthcare System, 16111 Plummer Street, North Hills, CA 91343 USA; 3 RAND Corporation, 1776 Main Street, 90401 Santa Monica, CA USA; 4 Veterans Affairs New York Harbor Healthcare System, 423 East 23rd Street, 10010 New York, NY USA; 5 New York University Langone Medical Center, 227 East 30th Room 642, 10016 New York, NY USA; 6 Department of Primary and Community Care, Radboud University Medical Center, Postbus 9101, 6500 HB Nijmegen, The Netherlands; 7 Veterans Affairs Greater Los Angeles Healthcare System, 11301 Wilshire Boulevard, 90073 Los Angeles, CA USA

**Keywords:** Cigarette smoking, Prevention, Schools, Systematic review, Girls

## Abstract

**Background:**

The purpose of this review is to study the effect of school-based interventions on smoking prevention for girls.

**Methods:**

We performed a systematic review of articles published since 1992 on school-based tobacco-control interventions in controlled trials for smoking prevention among children. We searched the databases of PubMed, Embase, Web of Science, The Cochrane Databases, CINAHL, Social Science Abstracts, and PsycInfo. Two reviewers independently assessed trials for inclusion and quality and extracted data. A pooled random-effects estimate was estimated of the overall relative risk.

**Results:**

Thirty-seven trials were included, of which 16 trials with 24,210 girls were included in the pooled analysis. The overall pooled effect was a relative risk (RR) of 0.96 (95 % confidence interval (CI) 0.86-1.08; *I*^2^=75 %). One study in which a school-based intervention was combined with a mass media intervention showed more promising results compared to only school-based prevention, and four studies with girl-specific interventions, that could not be included in the pooled analysis, reported statistically significant benefits for attitudes and intentions about smoking and quit rates.

**Conclusions:**

There was no evidence that school-based smoking prevention programs have a significant effect on preventing adolescent girls from smoking. Combining school-based programs with mass media interventions, and developing girl-specific interventions, deserve additional study as potentially more effective interventions compared to school-based-only intervention programs.

**Systematic review registration:**

PROSPERO CRD42012002322

**Electronic supplementary material:**

The online version of this article (doi:10.1186/s13643-015-0082-7) contains supplementary material, which is available to authorized users.

## Background

Worldwide, about 80–100,000 young people become addicted to tobacco every day [1], and smoking behavior established during adolescence often becomes a lifelong habit. In fact, 88 % of adult smokers who smoke daily report that they started smoking before the age of 18 [2].

Evidence supports that the motivation to start smoking at a young age differs between boys and girls. In a European study with 4000 adolescents, girls experiencing higher social pressure to smoke from friends were more likely to start smoking, but it was not a significant factor for boys [3]. Girls who had a favorite movie star who smoked on-screen had a greater increase in smoking initiation during adolescence, yet this relationship was not as strong for boys [4]. There is increasing evidence that the tobacco industry is focusing its efforts on the marketing of tobacco to women globally [5] by selling pink-colored cigarette packages that resemble perfume or lipstick boxes to make them attractive for girls. Industry analysts attributed the sharp increase of market share in 2007 of Camel cigarettes to the innovative fashion-themed Camel No. 9 marketing campaign aimed at young women that was launched in February 2007 [6]. The tobacco industry’s successful use of gender-based marketing to promote smoking provides a strong rationale for investigating gender-specific approaches to preventing smoking.

Helping children to not start smoking is a worldwide endorsed public health goal, and school-based programs are most commonly used to prevent smoking in children. However, most school-based interventions target boys and girls in the same, universal program. The World Health Organization Framework Convention on Tobacco Control (WHO FCTC) acknowledges gender-specific risks and the need for gender-specific strategies for more effective tobacco control [7]. In this systematic review, the authors focused on the effect of school-based interventions in smoking prevention for girls. The primary aim of this review was to determine how effective school-based interventions are in preventing smoking in girls, and the secondary objective was to determine which interventions are most successful. To answer these questions, studies were included that reported data on the effect of an intervention for girls, regardless of whether or not the intervention was girl-specific.

## Methods

This study was part of a larger project that was a scoping review of interventions outside the doctor’s office designed to prevent smoking and promote smoking cessation in women and girls. The study protocol is registered in PROSPERO (CRD Register 2012: CRD42012002322) [8]. The eligibility criteria for inclusion in the scoping review are reported in the protocol but, in general, were very broad-based, aimed at identifying smoking prevention and cessation interventions delivered outside the clinician’s office. There were few restrictions other than that publications had to be in the English language, published since 1992, and did not include treatment within a clinician’s office or certain specialized environments such as addiction-treatment facilities.

The data were collected through 2015 and analyzed in 2015.

### Data sources and searches

We conducted a review of key terms related to smoking cessation from PubMed, Embase, Web of Science, the Cochrane Databases in addition to the CINAHL, Social Science Abstracts, and PsycINFO from 1 January 1992 to 22 January 2015 and included articles published only in English (see Additional file 1: Table S1). We excluded letters to editors and editorials.

### Study selection

Two reviewers (MK and MF) independently reviewed the titles, and any title selected by either reviewer was accepted to the next stage. The two reviewers independently screened abstracts and full text articles against the inclusion and exclusion criteria using the DistillerSR software [9] and documented the reason for exclusion. Disagreements on inclusion were resolved by group consensus.

Based on the combination of policy-relevance and the availability of controlled trials, the authors narrowed the focus for the meta-analysis to school-based interventions for children with outcome data on smoking behavior for girls. Hence the inclusion criteria were as follows: population, children (less than 18 years of age at baseline) at school; intervention, any school-based tobacco-control intervention with the intention to prevent smoking or promote smoking cessation; comparison, including studies comparing outcomes of intervention groups to controls; design, trials (randomized, other controlled trials) and outcome: included studies that present smoking behavior results for girls. Smoking behavior was defined as changes in smoking behavior (quit smoking, intention to quit, retention rates) or intermediate outcome (changes in knowledge or attitudes toward smoking, satisfaction or acceptance of the intervention).

### Data extraction and quality assessment

For each study, we extracted the year, country where the study took place, funding source, age and ethnicity of the participants, study design, the number of participants, the intervention content, a description of the person or persons who delivered the intervention, the duration of the intervention, the duration of follow-up, and the smoking behavior outcome data.

Smoking behavior at baseline was coded as nonsmoker or smoker (daily, weekly, or monthly smoking). For non-smokers, outcome data were coded as started smoking versus never started smoking for non-smokers and quit smoking or did not quit smoking for smokers. We combined both outcomes when present, counting all smokers at follow-up (including new smokers and continuing smokers). We excluded studies that only reported intention to quit or intermediate outcomes from the pooled analysis.

The content or goals of the interventions were characterized into the following four categories: (1) gaining knowledge, (2) gaining more skills, (3) multiple strategies based at the school (i.e., quit-and-win contests, poster contests, displays, health fairs), and (4) multi-component programs (the school program is part of a larger anti-smoking campaign strategy outside the school). We developed this categorization prior to data extraction based on existing literature reviews on school-based prevention programs [10–12]. Ultimately, the different interventions aimed at gaining knowledge and/or gaining more skills were evaluated together (Additional file 2: Table S2. Characteristics and results for interventions designed to gain knowledge and/or more skills), versus multiple strategy interventions that were only school-based (Additional file: 3 Table S3. Characteristics and results for school-based multiple strategy interventions) and multi-component programs (Additional file 4: Table S4. Characteristics and results of school-based interventions with multi-component programs). The control group was defined as usual care when the control group received either no intervention (or nothing was described for the control group), the standard health education curriculum, the tobacco education curriculum, or drug-abuse education curriculum normally used at the school, or a delayed intervention (control group on a waiting list for the intervention).

Data extraction was done independently in duplicate (by MK and MF) with adjudication in case of disagreement by a third author.

### Risk-of-bias (quality) assessment

Criteria for judging the risk of bias were used, adapted from van Tulder 2003 [13], Boutron et al, 2005 (CLEAR NPT) [14] and the Cochrane Handbook of Systematic Reviews of Interventions, Chapter 8 (Version 5.1, updated March 2011) [15]. Two authors (MK and MF) assessed four aspects of risk of bias, with adjudication in case of disagreement by a third author (PS). Each potential risk of bias was assessed to be either at low risk, unclear risk (if no or insufficient data were provided which could be judged to assess bias), or at high risk (study design or execution could cause over or underestimation of the intervention effect) for each of the following criteria: selection bias (biased allocation to interventions) due to inadequate generation of a randomized sequence; selection bias due to dissimilarity between the intervention and control group at baseline; detection bias due to knowledge of the allocated interventions by outcome assessors; attrition bias due to amount, nature, or handling of incomplete outcome data; and performance bias or fidelity.

### Data synthesis and analysis

To be included in the pooled analysis, the number of girls within the intervention and control groups had to be reported. Also the number of new girl smokers at follow-up in the intervention and control groups had to be specified in order to calculate the relative risks. In some studies, the number of non-smokers at baseline was not stated in the article. Given the age of this population, we assumed the smoking rate would be very low [16, 17] and therefore set the baseline smoking rate to zero for these studies. We also conducted sensitivity analyses by removing these two studies.

We calculated relative risks for each trial, intervention compared to the usual. For trials that had more than one intervention group, we selected the one that had the largest published effect for the pooled analysis. Since randomization was usually done at the school or classroom level, we adjusted our results for clustering. Cluster adjustments are made by calculating the effective sample size for each trial and intervention group [18]. The effect sample size is calculated by dividing the total sample size by the design effect. The design effect takes into account the average cluster size and the intraclass correlation. Based on previous work done among measures of adolescent smoking, we used an intraclass correlation of 0.01 to calculate the design effect [19].

A pooled random-effects estimate [20] was estimated of the overall relative risk, and we performed a test of heterogeneity using *I*^2^ [21], where values of *I*^2^ close to 100 % represent high degrees of heterogeneity. We used the Begg rank correlation [22] and Egger regression asymmetry test [23] to test for evidence of publication bias. Publication bias occurs when the publication of results depends on their direction and significance [24]. Effects of quality were assessed by testing for differences within each of the five risk-of-bias measures. We used Stata 12 to conduct all analyses [25].

## Results

### Description of included studies

**Fig. 1:**
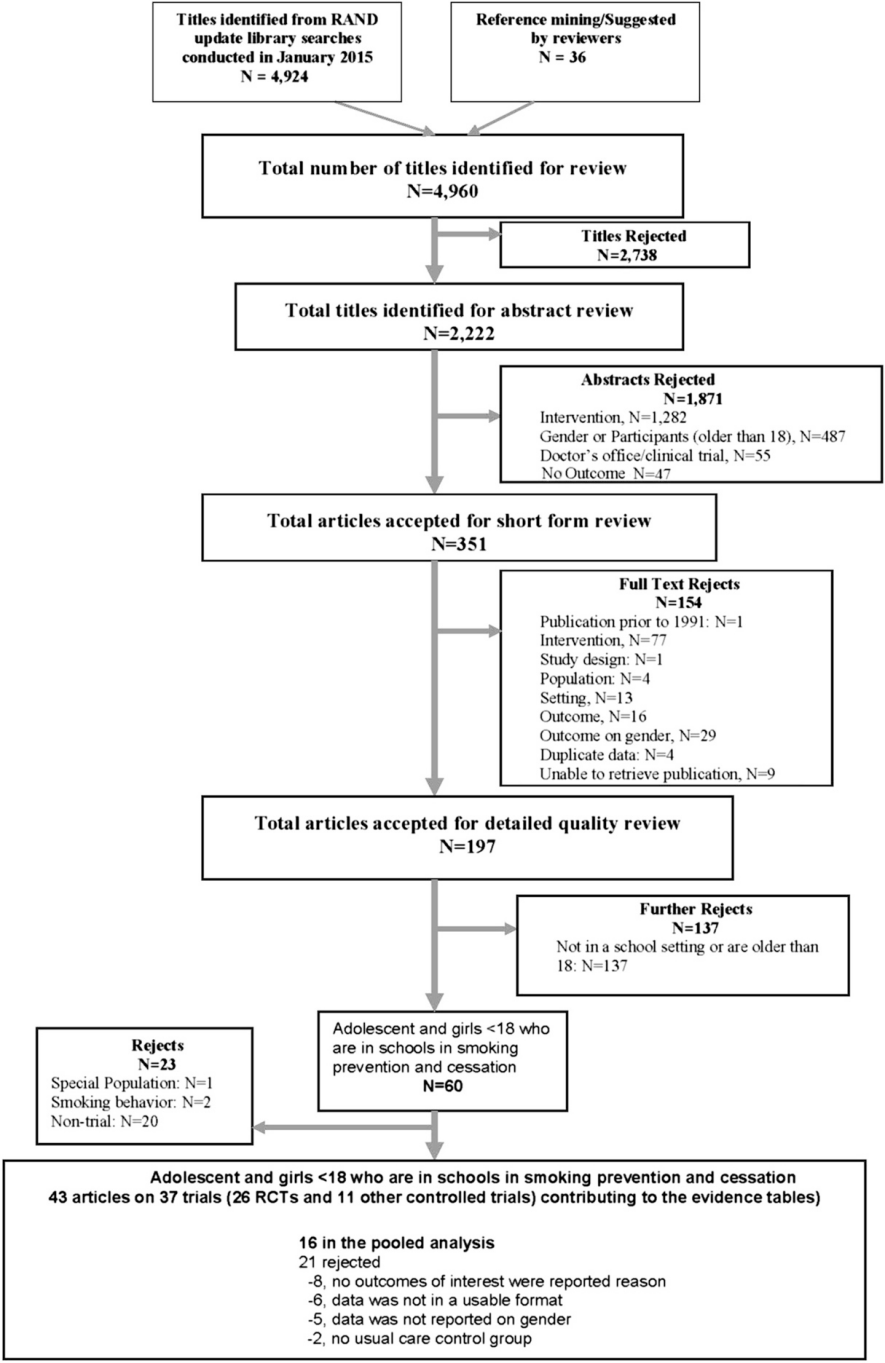
Literature flow

We reviewed 4924 titles from the electronic search and an additional 36 titles from occasional reference mining. Of the 4960 titles, 197 studies met the inclusion criteria after a title, abstract, and full article review (see Fig. 1). After further review, 37 controlled trials described in 43 articles were included in our data synthesis [16, 17, 26–60, 64, 70–74]; 26 randomized controlled trials and 11 other controlled trials; including 45 intervention groups and 38 control groups; giving a total of 83 arms, including girls from 20 different countries. Funding sources where all related to local or national governmental financing. Ethnicity was not reported in most articles. Full details of the included studies are shown in the evidence tables (Additional file 2: Table S2, Additional file 3: Table S3, Additional file 4: Table S4).

Nineteen study-arms had interventions focused on gaining knowledge and gaining more skills (e.g., peer-assisted learning, harm-minimization curriculum, take-charge-of-your-life program); six arms focused on gaining knowledge (e.g., informational lessons, social normative interventions); five arms focused on gaining more skills (e.g., refusal skills, life-skills training, good-behavior game); five arms had interventions based on multiple strategies (e.g., smoke-free class competition, school smoking-free policies); and ten arms had interventions consisting of multi-component programs (school interventions combined with mass media (anti-smoking advertisements) and/or community activities, parental interventions).

Only four studies included gender-specific interventions for girls [36, 50, 54, 58], while all others included the same intervention for boys and girls. Twenty-five studies had interventions mainly aimed at smoking prevention and/or cessation. In ten studies, the intervention was aimed at the prevention or reduction of substance abuse in general, whereas one study was aimed at good health [54] and one study at prevention of disruptive behavior [16].

Most trials were comparisons of an intervention group to a control group that received usual care (23 studies), and in two studies, the control group received a delayed intervention. In one study, the control group received the standard health education curriculum, and in four studies, the control group received the tobacco- or drug-abuse education curriculum normally used at the school, and in four studies, other activities took place. In three studies, two different interventions were compared to each other, and the school-based intervention was the same for the intervention and the control group [50, 51, 56]. Interventions were performed mostly by teachers, peers, coaches, and sometimes, parents or trainers outside the school were involved.

### Description of the excluded studies

One hundred thirty-seven studies were excluded that were not school-based or the participants where aged 18 or older (Fig. 1). Of the 60 articles that went on to detailed abstraction, one study was excluded because it was based on a special population [61], and two studies were excluded because there was no outcome reported on smoking behavior [62, 63]. Study designs that were not trials were excluded (*n* = 20).

### Effectiveness of the intervention

In the first category, interventions on gaining knowledge and/or gaining more skills (Additional file 2: Table S2), only one study showed significant positive results for the intervention for girls. This was a controlled trial [49] from Australia with 122 girls who were randomized to either a peer-led smoking-education program, a teacher-led smoking-education program, or a control group that received no intervention. The girls in the teacher-led program showed significant decreased risk of smoking after a seven-year follow-up (RR = 0.57; 95 % CI; 0.33–0.99) compared to girls in the control group. There was also one study showing a significant negative effect of the intervention—indicating that the control group was more effective in preventing smoking; it was a randomized clinical trial (RCT) [17] from the USA with a two-year school-based substance-abuse prevention program delivered by police officers to children. Data were analyzed from the five-year follow-up on 5814 girls and found a significantly higher risk of smoking for girls in the intervention group (RR = 1.3; 95 % CI; 1.21–1.41) compared to girls who received no intervention.

No studies reported a significant effect for girls for school-based interventions with multiple strategies (Additional file 3: Table S3).

In the third category, studies that included multi-component interventions (Additional file 4: Table S4), two studies had relative risks below 1, but the differences were not statistically significant [46, 47]. One of these, the Ariza et al. study, was a non-randomized controlled trial [47] with 575 girls from Spain. The school-based intervention consisted of the following three components: teacher-led lessons in year 1 and booster lessons in years 2 and 3, reinforcement of a smoke-free school policy with teacher training, and a community intervention targeting parents, leisure-time supervisors, sports organizations, and coaches. The control group did not receive any intervention. Although the relative risk for the intervention versus control was 0.82 at 3-year follow-up, the effect was not statistically significant (95 % CI; 0.64–1.03).

The Vartiainen et al. [46] study enrolled 1279 girls in a randomized controlled trial that included 14 information lessons about smoking and refusal skills training over a three-year period, and smoking prevention was integrated into regular subjects (e.g., math, Finnish, geography). Students hung up self-made anti-smoking posters, received newsletters where young people described their ways of refusing smoking, and were invited to participate in no-smoking competitions. The community-element of the program included parish confirmation camps, parents, and dentists. The schools in the control group received the standard health education curriculum. The risk ratio for the girls in the intervention group was 0.91 but not statistically significant (95 % CI; 0.82–1.00).

Two other multi-component studies [51] where excluded from the pooled analysis because the control group did not receive usual care. The first study [16] presented data on 10,170 girls from four different cities in the USA who received a 4-year school-based prevention curriculum with or without a mass media campaign. At the 5-year follow-up, the relative risk for girls to start smoking in the mass media group was 0.74 (95 % CI; 0.47–1.18) compared to those who only received the school-based prevention curriculum. The other study was a controlled trial among 1266 girls age 9 to 12 with a 4-year multi-component program including a mass media component and a school smoking-prevention program targeted primarily at girls. At the 6-year follow-up, the relative risk for girls to start smoking in the mass media group was 0.56 (95 % CI; 0.33-0.96) compared to the school-based prevention curriculum only.

### Pooled analysis

**Fig. 2:**
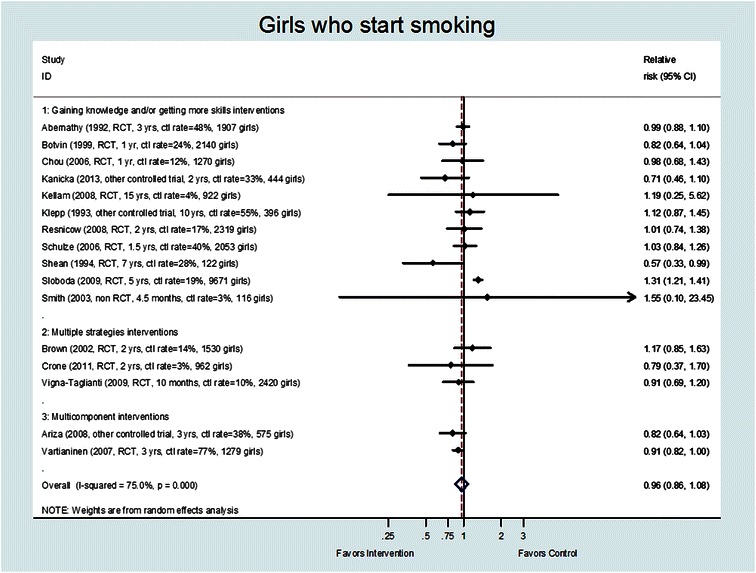
Pooled analysis

Twenty-one studies were excluded from the pooled analysis for the following reasons: the outcome of interest (percent of starters) was not reported (eight studies) [28, 29, 36, 45, 54, 57–59], the data was not reported by gender (five studies) [27, 30, 39, 53, 60], the data was not in a usable format—the number of girls who started smoking was not reported by intervention group (six studies) [32, 34, 35, 42, 52, 56], or they compared a mass media intervention combined with school intervention versus a school intervention alone, so their control group was not usual care (two studies) [50, 51]. Therefore, of the 37 included studies (described in 43 articles), 16 were analyzed in the pooled analysis [16, 17, 26, 31, 33, 37, 38, 40, 41, 43, 44, 46–49, 55] (see Fig. 2. Pooled analysis) including data on 24,210 girls.

**Fig. 3:**
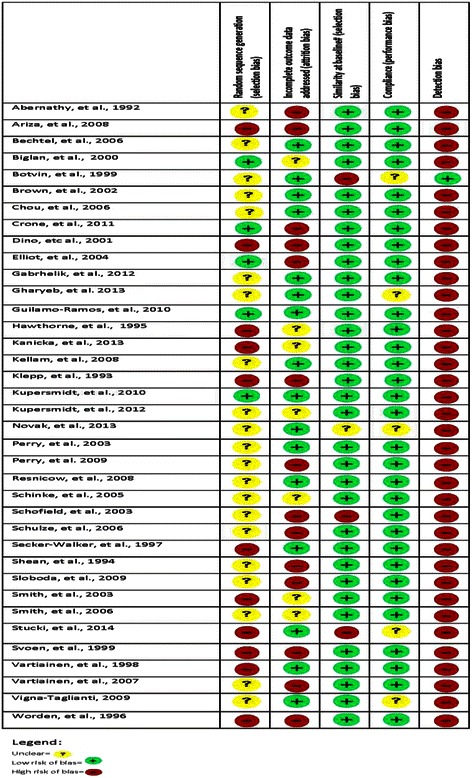
Risk of bias

In Fig. 3 (Risk of bias) the risk of bias of all 37 studies is presented. Regarding risk of bias, all included studies in the pooled analysis except one [31] were judged to be at high risk of detection bias (due to the use of self-report of smoking status as the outcome), and all except three [27, 30, 31] were judged to be at low risk for bias due to group similarity at baseline and compliance. Thirty-two studies were judged to be high or unclear risk of bias due to the sequence generation and twenty-one studies were judged to be at high or unclear risk of bias due to incomplete outcome data.

The percentage of girls smoking in the control group at the end of follow-up varied widely from 3 % [40, 44] to 77 % [46] in the studies included in the pooled analysis. Two of the 16 studies included in the pooled analysis (Fig. 2) had statistically significant differences between groups, one favoring the intervention [49] and the other favoring usual care [17]. The overall pooled effect was a RR = 0.96 (95 % CI; 0.86-1.108; *I*^2^ = 75 %). There was no indication of publication bias (Begg’s *p* = 0.558 and Egger’s *p* = 0.111). Testing for differences in effect based on quality/risk of bias was done, and no effect was found.

We imputed the baseline sample size for two studies that did not specify the number of non-smokers at baseline; we set the number of smokers at baseline to be zero. Results of the sensitivity analysis that excluded those studies with imputed baseline rate yielded a slightly lower relative risk and a more narrow confidence interval (pooled RR = 0.94, 95 % CI; 0.88–1.00; *I*^2^ = 6.0 %) than the pooled analyses with all 16 studies.

Of the 37 studies included, only four studies had gender-specific interventions for girls [36, 50, 54, 58], but then had to be excluded from the pooled analysis. Three of these gender-specific studies were excluded because the outcome of interest (smoking behavior) was not reported [36, 54, 58]. The fourth study was excluded because the control group received an intervention as well, so the intervention could not be compared to usual care [50]. However, all four of these gender-specific studies individually reported statistically significant positive effects. In the first study, 40 sport teams from 18 high schools based in two states (USA) including 928 girls were enrolled. Of these, 457 girls received the intervention called “ATHENA”: Athletes Targeting Health Exercise and Nutrition Alternative. The sports-coach and peers delivered the intervention in eight weekly 45-minute sessions that were incorporated into a team’s usual practice activities. Ten weeks after the start of the program, girls in the intervention group were less likely to report intentions toward future tobacco use than girls in the control group, and this difference was statistical significant (*p* < 0.05) [54]. In the second study, 91 girls from two high schools in New York, USA, received either a gender-specific individual computer intervention based on stress reduction or a group-based gender-neutral conventional substance-abuse prevention program. Two weeks after the intervention, girls who received the gender-specific intervention reported lower approval of cigarettes (*p* < 0.04), a lower likelihood of cigarette use if asked to do so by a best friend (*p* < 0.038), and stronger plans to avoid cigarettes in the next year (*p* < 0.046), than girls who received the conventional intervention [36]. In the third study, 5448 students were included from two matched pair communities in metropolitan areas in the USA. Within each matched pair, one community received a 4-year school-based intervention while the other received a mass media campaign along with the school-based intervention. The mass media campaign was especially focused on high-risk girls. Six years after the start of the study, the number of girls that had started smoking in the mass media group was lower than in the school-based intervention only group (a RR of 0.56 was calculated; 95 % CI; 0.33-0.96) [50]. In the fourth study [58], 188 girls received the not-on-tobacco intervention in ten 50-minute sessions over 10 weeks. The intervention was delivered in same-gender groups by same-gender facilitators, and the matched control schools were given only a brief intervention equal to a normal school curriculum on tobacco. At the seven-month follow-up, the quit rate for girls in the intervention group was almost four times higher than for the control group, and this difference was statistically significant (*p* = 0.043) [64].

## Discussion

In this systematic literature review, we examined the effect of school-based smoking-prevention interventions for girls. The pooled results of 16 studies provided no evidence of an effect (RR = 0.96) of the school-based interventions on smoking behavior outcomes for girls. However, the 95 % CI (0.86–1.08) does not exclude a modest-sized effect in magnitude similar to other smoking-control interventions [65].

There is a signal that interventions that include a mass media component may be more effective than school-based interventions alone; one study comparing a school-based intervention with a multi-component program showed a strong effect of the added mass media components [50]. Mass media interventions combined with school-based interventions are probably modifying the effect of the school-based intervention.

The only study that showed a significant effect [49] was a study among grade 7 students (age 12–13) from Australia, an age at which children and especially girls are highly influenced by peers [66].

Given that most of the studies included in this pooled analysis included a high percentage of smoking girls in the control group at follow-up, interventions with a small effect are clinically relevant with such high percentages of smoking girls. With the high percentages of smokers in a large part of the western world and the rapidly increasing smoking rates in parts of the eastern world, modest intervention effects for smoking prevention and cessation could have a serious impact globally.

### Gender-specific interventions

The literature on specific factors that influence a girl’s motivation to start smoking like social pressure and the influence of role models [3, 4] provide support that gender-specific interventions could be more effective than gender-neutral interventions at reducing smoking in girls. The four studies of girl-specific interventions [36, 50, 54, 58] that were identified, although limited in terms of either sample size, outcome measures, timing of the outcome, or the comparison group, provide some support for this. The amount to which girls are influenced by social pressure, movie stars, and the tobacco industry marketing also support that multi-component interventions with a mass media component could be more effective in girls compared to only school-based interventions. There were not enough multi-component interventions, and the number of included girls in these interventions was too small to adequately address this potential effect.

### Results of other reviews in this field

A recent Cochrane review [12] on school-based interventions concluded that there was an effect of smoking-prevention interventions for all children only when follow-up was greater than one year (OR = 0.52; 95 % CI; 0.30–0.88). The authors also looked at the effects by gender and found no statistically significant effects of the intervention for females (OR = 0.82; 95 % CI; 0.67–1.00) after a follow-up greater than one year. For a shorter follow-up (up to one year) they did find a modest effect (OR = 0.69; 95 % CI; 0.49–0.96) in girls. This result is in line with the results of the review. More studies were included in this pooled analysis [15] compared to the Cochrane review [7]. The five important differences between this review and the Cochrane review were [1] the scope of the review (Cochrane general scope with a subset analysis, this review scoped only girls) [2], publication date of the studies (studies published before 1992 were excluded in this review) [3], the stratification of follow-up time in up to one year and longer than one year (Cochrane) [4], the exclusion of studies without the number of non-smoking girls at baseline (Cochrane) [5], and the inclusion of non-randomized trials (Cochrane-only RCT). Therefore, our review adds evidence to the Cochrane review, which only included a limited number of randomized studies.

In another review of community interventions [67], most of the 25 trials combined the intervention with school-based interventions. Nine studies showed a significant long-term effect of the interventions, with two trials showing an intervention that was only effective in boys. In another review on the effectiveness of mass media campaigns in young people, three out of seven studies concluded that mass media interventions reduced the smoking behavior of young people [68]. One of the three studies was a media campaign aimed primarily at girls and found that the increase in the proportion of females smoking was significantly lower (8.6 %) in the intervention county than in the control county (12.4 %).

### Limitations of this review

This systematic review had several limitations. Only studies in which data on girls were included were presented. Out of the 37 studies, 11 studies had to be excluded because of missing or insufficient data on girls. This may have introduced presentation bias if authors were more likely to report the results by gender if such differences were actually seen. Given the overall ineffectiveness of the interventions, such bias, if present, would likely be small. In most studies, we considered the risk of detection bias as high, because of the self-report of smoking behavior. However, a recent study found that the validity of self-report in a large population including adolescents was high, with a sensitivity of 90 % [69], indicating that detection bias may not be a large risk. Attrition bias is a real problem in the studies, especially in studies with a long follow-up time. Longer follow-up often means higher attrition, and this most likely leads to overestimation of the effect due to the higher attrition of smokers compared to non-smokers. There was no difference in effect between the studies with a high or unclear risk and the studies with a low risk of attrition bias. Our search was limited to articles published in English. We also limited our search to articles from 1992 and onwards because sociological phenomena like smoking behavior change over time, and we judged that data from studies published before 1992 would be less relevant for today. The decision to focus this review on school-based interventions in the second stage of the search, and the difficulty in using well-defined search terms for this review, could have resulted in missing school-based studies that did not focus on girls specifically. However, our search should have captured most studies that identified gender-specific results. We did not search for ongoing studies through online clinical trial registries or contact authors for this review.

When the percentage of smokers is low, it is less likely that an intervention shows a significant effect in reducing smoking rates. In the pooled analysis, only three studies were included with a low percentage (<5 %) of smokers in the control group at the end of follow-up (see Fig. 2). Therefore it is not likely that the result was due to a low baseline risk of smoking in the included studies. The control group in the included studies was not well described in most studies, or consisted of a usual tobacco or health education curriculum. Therefore, it is not possible to know if there was enough contrast in the programs delivered to the control and intervention groups, which perhaps partially explains why there was no significant result of the interventions. An additional limitation is that we grouped together studies that included smoking prevention as one part of a substance-use program with studies that were focused solely on smoking prevention. It may be that focused studies will show more benefit; although, we did not find evidence of that in our review.

In studies that included more than one active intervention arm, we analyzed the comparison that had the greatest effect in the pooled analysis. This approach is described in the Cochrane handbook as a way to overcome a unit-of-analysis error, and the resulting bias will be toward showing a greater effect. Our results show “no effect”; therefore, the substitution of any of the other active intervention arms would only lessen the effect. An additional limitation is the degree of heterogeneity among studies, which means that pooled results should be interpreted with caution. Part of the reason of this heterogeneity could be the pooling of studies reporting “smoking behavior” in a variety of different ways.

## Conclusions

In summary, in a systematic review of the literature and meta-analysis, no evidence was found that gender-neutral school-based smoking-prevention programs have a significant effect on preventing teen-aged girls from smoking, although we could not exclude a modest effect due to the large confidence intervals. A modest effect could still be of potential public health significance. We urge researchers in this field to routinely report the effect of the intervention for boys and girls of gender-neutral smoking-prevention interventions so we can sharpen these estimates. We found a signal that suggests that adding mass media campaigns to gender-neutral school-based interventions might increase their effectiveness, pointing to a need for future research in these areas. Lastly, a few studies suggest that it may be a more successful strategy to develop girl-specific interventions that target the reasons on why girls start smoking.

## Additional files

Additional file 1: Table S1. Literature search methodology.
Additional file 2: Table S2. Evidence tables for prevention of smoking: interventions on gaining knowledge and/or more skills.
Additional file 3: Table S3. Evidence tables with studies on school-based interventions with multiple strategies.
Additional file 4: Table S4. Evidence tables with studies on school-based interventions with multi-component programs.

## Abbreviations

CI: confidence interval
RCT: randomized controlled trial
RR: relative risk
WHO FCTC: World Health Organization Framework Convention on Tobacco Control
