# Supplementary material for: Systematic review of school-based interventions to prevent smoking for girls
Source: Syst Rev. 2015 Aug 14;4:109. doi: 10.1186/s13643-015-0082-7 (PMC4536766; doi:10.1186/s13643-015-0082-7)
Supplement: Additional file 1: Table S1. — Literature search methodology. [file 13643_2015_82_MOESM1_ESM.docx]

**Additional file 1, Table 1. Literature search methodology**

**Search #1:**

**DATABASE SEARCHED AND TIME PERIOD COVERED**

PubMed

01/01/92– 01/22/2015

Language: English

(Women OR woman OR female OR females OR girl OR girls OR gender OR male OR males OR man OR men OR boys OR pregnan*)[ti/ab]

AND

(Smoking OR tobacco OR smoker OR smokers)[ti/ab]

AND

Campaign* OR communicat* OR marketing OR advertis* OR media OR prevention program*

AND

Cessation OR intervention or intervene? OR  quit* OR prevent*

AND females[MESH]

AND human[MESH]

NOT letter[pt] OR editorial[pt]

==================================================================================

**Search #2:**

**DATABASE SEARCHED AND TIME PERIOD COVERED**

PubMed

01/01/92– 01/22/2015

Language: English

Women OR woman OR female OR females OR girl OR girls OR gender OR male OR males OR man OR men OR boys OR pregnan*

AND

Smoking OR tobacco OR smoker OR smokers

AND

Campaign* OR communicat* OR marketing OR advertis* OR media OR prevention program*

AND

Cessation OR intervention or intervene? OR quit* OR prevent*

AND

in process [sb] or publisher [sb]

==================================================================================

**SEARCH #3**

**DATABASE SEARCHED AND TIME PERIOD COVERED**

Embase

01/01/92– 01/22/2015

Language: English

Limit: With abstracts

Limit: humans

Search Strategy:

(Women:ti OR woman:ti OR female:ti OR females:ti OR girl:ti OR girls:ti OR gender:ti OR male:ti OR males:ti OR man:ti OR men:ti OR boys:ti OR pregnan*:ti) OR (Women:ab OR woman:ab OR female:ab OR females:ab OR girl:ab OR girls:ab OR gender:ab OR male:ab OR males:ab OR man:ab OR men:ab OR boys:ab OR pregnan*:ab)

AND

(Smoking:ti OR tobacco:ti OR smoker:ti OR smokers:ti) OR (Smoking:ab OR tobacco:ab OR smoker:ab OR smokers:ab)

AND

Campaign* OR communicat* OR marketing OR advertis* OR media OR prevention program OR prevention programs

AND

Cessation OR intervention or intervene? OR quit* OR prevent*

NOT

Editorial:it OR letter:it

**=============================================================================**

**SEARCH #4A**

**DATABASE SEARCHED AND TIME PERIOD COVERED**

Cochrane: Reviews

01/01/92– 01/22/2015

No language limiter

(Women:ti OR woman:ti OR female:ti OR females:ti OR girl:ti OR girls:ti OR gender:ti OR male:ti OR males:ti OR man:ti OR men:ti OR boys:ti OR pregnan*:ti) OR (Women:ab OR woman:ab OR female:ab OR females:ab OR girl:ab OR girls:ab OR gender:ab OR male:ab OR males:ab OR man:ab OR men:ab OR boys:ab OR pregnan*:ab)

AND

(Smoking:ti OR tobacco:ti OR smoker:ti OR smokers:ti) OR (Smoking:ab OR tobacco:ab OR smoker:ab OR smokers:ab)

AND

Campaign* OR communicat* OR marketing OR advertis* OR media OR prevention program OR prevention programs

AND

Cessation OR intervention or intervene? OR quit* OR prevent*

NOT

Editorial:it OR letter:it

**Number of results: 14**

=================================================================================

**SEARCH #4B**

**DATABASE SEARCHED AND TIME PERIOD COVERED**

Cochrane: Reviews, Other Reviews, Trials, Methods Studies, Technology Assessments, Economic Evaluations, Groups

01/01/92– 01/22/2015

No language limiter

(Women:ti OR woman:ti OR female:ti OR females:ti OR girl:ti OR girls:ti OR gender:ti OR male:ti OR males:ti OR man:ti OR men:ti OR boys:ti OR pregnan*:ti) OR (Women:ab OR woman:ab OR female:ab OR females:ab OR girl:ab OR girls:ab OR gender:ab OR male:ab OR males:ab OR man:ab OR men:ab OR boys:ab OR pregnan*:ab)

AND

(Smoking:ti OR tobacco:ti OR smoker:ti OR smokers:ti) OR (Smoking:ab OR tobacco:ab OR smoker:ab OR smokers:ab)

AND

Campaign* OR communicat* OR marketing OR advertis* OR media OR prevention program OR prevention programs

AND

Cessation OR intervention or intervene? OR quit* OR prevent*

NOT

Editorial:it OR letter:it

=================================================================================

**SEARCH #5**

**DATABASE SEARCHED AND TIME PERIOD COVERED**

Web of Science

01/01/92– 01/22/2015

Language: English

TS=(Women OR woman OR female OR females OR girl OR girls OR gender OR male OR males OR man OR men OR boys OR pregnan*)

AND

TS=(Smoking OR tobacco OR smoker OR smokers)

AND

TS=Campaign* OR communicat* OR marketing OR advertis* OR media OR prevention program OR prevention programs

AND

TS=(Cessation OR intervention or intervene? OR  quit* OR prevent*)

AND NOT

DT=(editorial material or letter)

==================================================================================

**SEARCH #6**

**DATABASE SEARCHED AND TIME PERIOD COVERED**

PsycInfo

01/01/92– 01/22/2015

Language: English

NOTE: EBSCO databases – difficult to exclude letters and editorials; removal done via searches for those words in the EndNote file

TI: (Women OR woman OR female OR females OR girl OR girls OR gender OR male OR males OR man OR men OR boys OR pregnan*) OR TI: (Smoking OR tobacco OR smoker OR smokers)

AND

AB: (Women OR woman OR female OR females OR girl OR girls OR gender OR male OR males OR man OR men OR boys OR pregnan*) OR AB: (Smoking OR tobacco OR smoker OR smokers)

AND

Campaign* OR communicat* OR marketing OR advertis* OR media OR prevention program OR prevention programs

AND

Cessation OR intervention or intervene? OR  quit* OR prevent*

=================================================================================

**SEARCH #7**

**DATABASE SEARCHED AND TIME PERIOD COVERED**

CINAHL

01/01/92– 01/22/2015

Language: English

NOTE: EBSCO databases – difficult to exclude letters and editorials; removal done via searches for those words in the EndNote file

TI: (Women OR woman OR female OR females OR girl OR girls OR gender OR male OR males OR man OR men OR boys OR pregnan*) OR TI: (Smoking OR tobacco OR smoker OR smokers)

AND

AB: (Women OR woman OR female OR females OR girl OR girls OR gender OR male OR males OR man OR men OR boys OR pregnan*) OR AB: (Smoking OR tobacco OR smoker OR smokers)

AND

Campaign* OR communicat* OR marketing OR advertis* OR media OR prevention program OR prevention programs

AND

Cessation OR intervention or intervene? OR  quit* OR prevent*

**==================================================================================**

**SEARCH #8**

**DATABASE SEARCHED AND TIME PERIOD COVERED**

Social Science Abstracts:

01/01/92– 01/22/2015

Language: English

NOTE: EBSCO databases – difficult to exclude letters and editorials; removal done via searches for those words in the EndNote file

TI: (Women OR woman OR female OR females OR girl OR girls OR gender OR male OR males OR man OR men OR boys OR pregnan*) OR TI: (Smoking OR tobacco OR smoker OR smokers)

AND

AB: (Women OR woman OR female OR females OR girl OR girls OR gender OR male OR males OR man OR men OR boys OR pregnan*) OR AB: (Smoking OR tobacco OR smoker OR smokers)

AND

Campaign* OR communicat* OR marketing OR advertis* OR media OR prevention program OR prevention programs

AND

Cessation OR intervention or intervene? OR  quit* OR prevent*
