# Supplementary material for: Systematic review of school-based interventions to prevent smoking for girls
Source: Syst Rev. 2015 Aug 14;4:109. doi: 10.1186/s13643-015-0082-7 (PMC4536766; doi:10.1186/s13643-015-0082-7)
Supplement: Additional file 2: Table S2. — Evidence tables for prevention of smoking: interventions on gaining knowledge and/or more skills. [file 13643_2015_82_MOESM2_ESM.docx]

**Additional file 2 Table 2. Characteristics and results for interventions designed to gain knowledge and/or more skills.**

| **Author, Year, Country, Study Design (Unit of Randomization)** | **No of Girls Enrolled** | **Age of Study Population** | **Interventions** | **One that performs the intervention** | **Duration of Intervention** | **Follow Up Time Since Baseline** | **RR girls (95% CI)** |
| --- | --- | --- | --- | --- | --- | --- | --- |
| Abernathy et. al, 1992(37)  Canada; RCT (School) | Intervention 1: Enrolled: 560, Final Sample: 395  Intervention 2: Enrolled: 379, Final Sample: 272  Control: Enrolled: 968, Final Sample: 694 | 11-12 | Intervention 1: Gain Knowledge, More Skills, PAL (peer assisted learning) - all lessons smoking prevention program*  Intervention 2: Gain Knowledge, More Skills, PAL (peer assisted learning) – student received less than 6 lessons  Control: No intervention/nothing described | Intervention 1: Teacher  Intervention 2: Teacher students received less than full intervention | 3 months  Number of Sessions: 6 | 3 years | 0.99 (0.88, 1.10) |
| Bechtel et. al, 2006(39)  Vicary et. al, 2006(70)  USA; RCT (School) | Intervention 1: Enrolled: 128  Intervention 2: Enrolled: 108  Control: Enrolled: 98 | 12-13 | Intervention 1: Gain Knowledge, More Skills, Botvin's Life Skills Training (LST) for substance abuse prevention  Intervention 2: Gain Knowledge, More Skills, integrated or infused delivery of Botvin's Infused Life Skills Training (I-LST)  Control: No intervention/nothing described | Intervention 1: Teacher  Intervention 2: Teacher | 3 years  Number of Sessions: 32 | 3 years | no usable data; data not reported by gender |
| Botvin et. al, 1999(31)  USA; RCT (School) | Intervention: Final Sample: 1242  Control: Final Sample: 898 | 11-13 | Intervention: Gain Knowledge, More Skills, social resistance skills, psychosocial program for substance abuse  Control: Five session information only drug curriculum | Intervention: Teacher  Control: Teacher | 2 years  Number of Sessions: 25 | 1 year | 0.82 (0.64, 1.04) |
| Chou et. al, 2006(38)  China; RCT (School) | Intervention: Enrolled: 636, Final Sample: 599  Control: Enrolled: 634, Final Sample: 607 | 12-14 | Intervention: Gain Knowledge, More Skills, smoking prevention curriculum social normative intervention  Control: Usual classroom program | Intervention: Trainer | 13 weeks  Number of Sessions: 13 | 1 year | 0.98 (0.68, 1.43) |
| Dino et. al, 2001(58)  Dino et. al, 2001(64)  USA; Other Controlled Trial | Intervention : NR  Control: NR | 14-19 | Intervention: Gain Knowledge, More Skills, NOT (not on tobacco) a gender sensitive teen smoking cessation program  Control: Brief intervention included normal class room curriculum | Intervention: Trainer | 10 weeks plus boosters (2-4 weeks)  Number of Sessions: 10 plus 4 boosters | 7.3 months | outcome of interest not reported (percentage of girls starting smoking) |
| Elliot et. al, 2004(54)  USA; RCT (School) | Intervention: Enrolled: 457, Final Sample: 337  Control: Enrolled: 471, Final Sample: 331 | 14-16 | Intervention: Gain Knowledge, More Skills, ATHENA (athletes targeting health exercise and nutrition alternative) gender specific intervention  Control: Preprinted pamphlets concerning disordered eating, drug use; and sports nutrition | Intervention: Peer, coach (sports) | 8 weeks  Number of Sessions: 8 | 10 weeks | outcome of interest not reported (percentage of girls starting smoking) |
| Gabrhelik et. al, 2012(34)  Czech Republic; RCT (School) | Intervention: Enrolled: 510  Control: Enrolled: 417 | 11-13 | Intervention: Gain Knowledge, More Skills, ‘Unplugged’ for substance abuse prevention  Control: No intervention/nothing described | Intervention: Teacher | 1 year  Number of Sessions: 12 | 3.3 years | no usable data; the number of girls who started smoking was not reported by intervention group |
| Ghrayeb et. al, 2013(29)  Palestine; RCT (School) | Intervention: Enrolled: 60  Control: Enrolled: 60 | 16-18 | Intervention: "Gaining knowledge; the compelling the challenge (CTC) curriculum, culturally tailored on dimensions of health behavior | Intervention: not reported | 8 weeks  Number of sessions: 5 | 3 months | Outcome of interest not reported: smoking behavior (only smoking related knowledge reported) |
| Hawthorne et. al, 1995(35)  Australia; Other Controlled Trial | Intervention: NR  Control: NR | 11-12 | Intervention: Gain Knowledge, More Skills, Life Education's drug education program  Control: Conventional school-based drug education program same hours as intervention program | Intervention: Teacher | 5 years  Number of Sessions: 15 | 5 years | no usable data; the number of girls who started smoking was not reported by intervention group |
| Kanicka et. al, 2013(33)  Poland; Other Controlled Trial | Intervention: Enrolled: 224  Control: Enrolled: 220 | 13 | Intervention: Gain Knowledge, More Skills, educational antitobacco and health program program  Control: No intervention/nothing described | Intervention: Trainer | 2 years | 2 years | 0.71 (0.46, 1.10) |
| Kellam et. al, 2008(16)  USA; RCT (School and Classroom) | Intervention: NR  Control 1: NR  Control 2: NR | 6-7 | Intervention: More Skills, GBG (good behavior game) socialize children to reduce aggressive, disruptive behaviors  Control 1: Internal control received no intervention within same school but other classroom  Control 2: External control receieved no intervention in other school | Intervention: Teacher | 2 years | 13-15 years | 1.19 (0.25, 5.62) |
| Klepp et. al, 1993(41)  Norway; Other Controlled Trial | Intervention: Enrolled: 204, Final Sample: 154  Control: Enrolled: 192, Final Sample: 133 | 10-13 | Intervention: Gain Knowledge, More Skills, Youth Study Smoking Prevention Program (YSSPR)  Control: No intervention/nothing described | Intervention: Peer | 15 months  Number of Sessions: 10 | 10 years | 1.12 (0.87, 1.45) |
| Kupersmidt et. al, 2010(57)  USA; RCT (School) | Intervention: Final Sample: 165  Control: Final Sample: 179 | 7-13 | Intervention: More Skills, Media Detective (media training) substance using prevention program  Control: Usual classroom program | Intervention: Teacher | 10 days  Number of Sessions: 10 | 2 weeks | outcome of interest not reported: percentage of girls starting smoking |
| Kupersmidt et. al, 2012(60)  USA; RCT (School) | Intervention: Enrolled: 130  Control: Enrolled: 103 | 11-14 | Intervention: More Skills, media ready program (prevention alcohol & tobacco abuse)  Control: Usual classroom program | Intervention: Teacher | 10 days  Number of Sessions: 10 | 2 weeks | no usable data; data not reported by gender |
| Novak et. al, 2013(28)  Czech Republic; RCT (School) | Intervention:  Enrolled: 525  Control: Enrolled: 425 | 11-12 | Intervention: Gaining knowledge, more skills, Unplugged prevention program.  Control: No intervention/nothing described | Intervention: Teacher | One academic year  Number of sessions: 12 | Multiple follow-ups; less then one year and between one and five years | Outcome of interest not reported: number of girls starting smoking |
| Resnicow et. al, 2008(48)  South Africa; RCT (School) | Intervention 1: Final Sample: 803  Intervention 2: Final Sample: 786  Control: Final Sample: 730 | 14-15 | Intervention 1: More Skills, life skills training curriculum for smoking prevention*  Intervention 2: More Skills, KEEP LEFT harm minimization curriculum for smoking prevention  Control: Usual classroom program including tobacco education curriculum | Intervention 1: Teacher  Intervention 2: Teacher | 2 years  Number of Sessions: 16 | 2 years | 1.01 (0.74, 1.38) |
| Schinke et. al, 2005(36)  USA; RCT (School) | Intervention: Final Sample: 47  Control: Final Sample: 44 | 12-13 | Intervention: Gain Knowledge, More Skills, gender-specified computer intervention prevention on substance abuse  Control: Usual classroom program including conventional drug abuse prevention program called “Keep a Clear Mind” | Intervention: Teacher | 20-30 minutes  Number of Sessions: 1 | 2 weeks | outcome of interest not reported: smoking behavior |
| Schulze et. al, 2006(55)  Germany; RCT (School) | Intervention: Enrolled: 1113  Final Sample: 510  Control: Enrolled: 940, Final Sample: 486 | 11-15 | Intervention: Gain Knowledge, More Skills, smoke-free class competition  Control: No intervention/nothing described | Intervention: Teacher | 6 months | 18 months | 1.03 (0.84, 1.26) |
| Shean et. al, 1994(49)  Australia; RCT (School) | Intervention 1: Final Sample: 36  Intervention 2: Final Sample: 45  Control: Final Sample: 41 | 12-12 | Intervention 1: Gain Knowledge, More Skills, peer led smoking education program*  Intervention 2: Gain Knowledge, More Skills, teacher led smoking education program  Control: No intervention/nothing described | Intervention 1: Teacher, peer  Intervention 2:  Teacher | 6 months  Number of Sessions: 5 | 7 years | 0.57 (0.33, 0.99) |
| Sloboda et. al, 2009(17)  USA; RCT (Community or school district) | Intervention: Enrolled: 5566, Final Sample: 3221  Control: Enrolled: 4105, Final Sample: 2593 | 12-13 | Intervention: Gain Knowledge, More Skills, ‘Take charge of your life’ (prevents use of alcohol, tobacco, marihuana)  Control: No intervention/nothing described | Intervention: D.A.R.E. police-officers | 2 years in a 3 year period  Number of Sessions: 12 | 5 years | 1.31 (1.21, 1.41) |
| Smith et. al, 2006(45)  USA; RCT (Classroom) | Intervention 1: Final Sample: 127  Intervention 2: Final Sample: 74  Control: Final Sample: 124 | 12-18 | Intervention 1: Gain Knowledge, Short term (ST) cosmetic content anti- smoking advertisements  Intervention 2: Gain Knowledge, Long Term (LT) health content anti smoking advertisements  Control: No intervention/nothing described | Intervention 1&2: Media TV/video | 3 weeks  Number of Sessions: 15 | 9 weeks | outcome of interest not reported: percentage of girls starting smoking |
| Smith et. al, 2003(44)  USA; Other Controlled Trial | Intervention 1: Final Sample: 40  Intervention 2: Final Sample: 33  Control: Final Sample: 43 | 14-18 | Intervention 1: Gain Knowledge, anti-smoking-advertisement Short Term (ST) cosmetic appeal  Intervention 2: Gain Knowledge, Long Term (LT) health appeal*  Control: No intervention/nothing described | Intervention 1&2: Advertisement television/print/internet | 4.5 months  Number of Sessions: 9 | 4.5 months | 1.55 (0.10, 23.5) |
| Svoen et. al, 1999(42)  Norway; Other Controlled Trial | Intervention: NR  Control: NR | 13-15 | Intervention: Gain Knowledge, contest, videos, posters, contract, anti-smoking lessons and media  Control: No intervention/nothing described | Intervention: Teacher, health visitor; physician | 3 years  Number of Sessions: 32 | 3 years | no usable data; the number of girls who started smoking was not reported by intervention group |

*Intervention with largest calculated effect, used in pooled analysis

**Legend:** ATHENA=Athletes Targeting Health Exercise and Nutrition Alternative; D.A.R.E=Drug Abuse Resistance Education; LST=Life Skills Training; LT=Long Term; NOT=Not On Tobacco; NR=Not Reported; PAL=Peer Assisted Learning; RCT=Randomized Controlled Trial; RR=Risk Ratio; ST=Short Term; YSSPR=Youth Study Smoking Prevention Program (YSSPR)
