# Supplementary material for: Systematic review of school-based interventions to prevent smoking for girls
Source: Syst Rev. 2015 Aug 14;4:109. doi: 10.1186/s13643-015-0082-7 (PMC4536766; doi:10.1186/s13643-015-0082-7)
Supplement: Additional file 3: Table S3. — Evidence tables with studies on school-based interventions with multiple strategies. [file 13643_2015_82_MOESM3_ESM.docx]

**Additional file 3 Table 3. Characteristics and results for school-based multiple strategies interventions**

| **Author, Year, Country, Study Design (Unit of Randomization)** | **No of Girls Enrolled** | **Age of Study Population** | **Interventions** | **One that performs the intervention** | **Duration of Intervention** | **Follow Up Time** | **RR girls (95% CI)** |
| --- | --- | --- | --- | --- | --- | --- | --- |
| Brown et. al, 2002(43)  Canada; RCT (School) | Intervention: Enrolled: 797  Control: Enrolled: 733 | 14-16 | Intervention: Multiple Strategies, smoking prevention curriculum  Control: Usual classroom program | Intervention: Teacher, peer, community participants | 2 years | 2 years | 1.17 (0.85, 1.63) |
| Crone et. al, 2011(40)  The Netherlands; RCT (School) | Intervention: Final Sample: 535  Control: Final Sample: 427 | 10-12 | Intervention: Multiple Strategies, smoking prevention lessons  Control: Usual classroom program | Intervention: Teacher | 2 years  Number of Sessions: 6 | 2 years | 0.79 (0.37, 1.70) |
| Perry et. al, 2009(32)  India; RCT (School) | Intervention: NR  Control: NR | 11-15 | Intervention: Multiple Strategies, Mobilizing Youth for Tobacco-Related Initiatives in India (MYTRI)  Control: Usual classroom program | Intervention: Teacher, peer, parent | 2 years  Number of Sessions: 13 | 2 years | no usable data; the number of girls who started smoking was not reported by intervention group |
| Stucki et. al,  2014(27)  Switzerland; other controlled trial (non randomized) | Intervention: Enrolled: 325  Control: Enrolled: 226 | 12-14 | Intervention: Multiple strategies, the winning of a prize; school class contract management; classmate peers as rolemodels; social norms  Control: No intervention/nothing described | Intervention: teacher, non smoking peers | 6 months | 7 months | No usable data, outcome not reported by gender |
| Vigna et. al, 2009(26) Spain, Germany, Belgium, Sweden, Greece, Austria, Italy; RCT (school) | Intervention: Final sample: 1497  Control: Final sample: 1538 | 11-18 | Intervention: Multiple strategies, “Unplugged’’ interpersonal and intrapersonal skills; in addition: 1/3 of schools seminars for the parents, 1/3 of schools peer-based intervention | Teacher, peers | 4 months  Number of sessions: 12 | 3 months | 0.91  (0.69, 1.20) |

**Legend:** MYTRI= Mobilizing Youth for Tobacco-Related Initiatives in India; NR=Not Reported; RCT=Randomized Controlled Trial; RR=Risk Ratio
