# Supplementary material for: Systematic review of school-based interventions to prevent smoking for girls
Source: Syst Rev. 2015 Aug 14;4:109. doi: 10.1186/s13643-015-0082-7 (PMC4536766; doi:10.1186/s13643-015-0082-7)
Supplement: Additional file 4: Table S4. — Evidence tables with studies on school-based interventions with multi-component programs. [file 13643_2015_82_MOESM4_ESM.docx]

**Additional file 4 Table 4. Characteristics and results of school-based interventions with multi-component programs**

| **Author, Year, Country, Study Design (Unit of Randomization)** | **No of Girls Enrolled** | **Age of Study Population** | **Interventions** | **One that performs the intervention** | **Duration of Intervention** | **Follow Up Time** | **RR girls (95% CI)** |
| --- | --- | --- | --- | --- | --- | --- | --- |
| Ariza et. al, 2008(47)  Spain; Other Controlled Trial | Intervention: Final Sample: 285  Control: Final Sample: 290 | 12-13 | Intervention: Multi-component program, European Smoking Prevention Framework Approach (ESFA) school free policy, parental intervention, community  Control: No intervention/nothing described | Intervention: Teacher, posters, school, tobacco sales controllers | 3 years  Number of Sessions: 17 | 3 years | 0.82 (0.64, 1.03) |
| Biglan et. al, 2000(53)  USA; RCT Community or school district | Intervention: NR  Control: NR | 12-15 | Intervention: Multi-component program to prevent tobacco use: Project Programs to Advance Teen Health (PATH); a school based prevention and community program  Control: School-based tobacco and other substance use prevention program | Intervention: Teacher, community coordinator; and youth and adult volunteers from community | 2.5 years  Number of Sessions: 5 | 5 years | no usable data |
| Guilamo-Ramos et. al, 2010(56)  USA; RCT (Individual) | Intervention: NR  Control: NR | 11-13 | Intervention: Multi-component: Raising Smoke-Free Kids  School-based smoking prevention intervention Towards No Tobacco (TNT) plus a mother add-on condition called Raising Smoke-Free Kids:  Control: TNT Intervention and mother received a control curriculum about how to choose a high school (youths in New York City can choose their high school). | Intervention: Teacher, mother  Control: Teacher | 6 months  Number of Sessions: 2 + 2 booster calls for the moms | 15 months | no usable data; the number of girls who started smoking was not reported by intervention group |
| Perry et. al, 2003(59)  USA; RCT (School) | Intervention 1: Final Sample: 1249  Intervention 2: Final Sample: 1254  Control: Final Sample: 1015 | 12-13 | Intervention 1: Gain Knowledge, More Skills, DARE (drug abuse resistance education)  Intervention 2: Multi-component program: DARE Plus on drug use and violence  Control: Usual classroom program | Intervention 1: Police officer  Intervention 2: Teacher, peer, police-officer and community members | 2 years  Number of Sessions: 10 | 2 years | outcome of interest not reported |
| Secker-Walker et. al, 1997(51)  Flynn et al, 1994(71)  Flynn et al, 1992 (72)  USA; Other Controlled Trial | Intervention: Final Sample: 5481  Control: Final Sample: 4689 | 10-13 | Intervention: Multi-component program on smoking, media, mass media + school program  Control: Smoking prevention curriculum only | Intervention: teacher, government  Control: teacher | 4 years  Number of Sessions: 23 | 5 years | 0.74 (0.47, 1.18) |
| Schofield et al, 2003(30)  Australia, RCT (School) | Intervention: Enrolled: 2573, Final sample 1007  Control: Enrolled 2268; Final sample 845 | 12-14 | Intervention: Multi-component Health Promoting Schools program (HPS) including community elements (tobacco retailers/parents)  Control: Usual classroom program, only given resources for non-smoking if requested | Intervention: Teacher, peer, liaison-officer | 2 years | 2 years | no usable data |
| Vartiainen et. al, 1998(52)  Finland; Other Controlled Trial | Intervention 1: NR  Intervention 2: NR  Control: NR | 12-13 | Intervention 1: Multi-component program: smoking prevention program by health educator+ community based adult cessation program  Intervention 2: Same as above except teacher led  Control: No intervention/nothing described | Intervention 1: Health educator; mass media; community, peer  Intervention 2: Teacher; mass media; community | 3 years  Number of Sessions: 10 | 15 years | no usable data; the number of girls who started smoking was not reported by intervention group |
| Vartiainen et. al, 2007(46)  Finland; RCT (School) | Intervention: Enrolled: 593, Final Sample: 453  Control: Enrolled: 686, Final Sample: 398 | 12-14 | Intervention: Multi-component program, information lessons, refusal skills training for smoking prevention  Control: Standard health education curriculum | Intervention: Teacher, school nurses; school dentists; camp leaders | 3 years  Number of Sessions: 14 | 3 years | 0.91 (0.82, 1.00) |
| Worden et. al, 1996(50)  Worden et al., (73)  Flynn et. al, 1995(74)  USA; Other Controlled Trial | Intervention: NR  Control: NR | 9-12 | Intervention: Multi-component program, mass media + school smoking prevention program targeted primarily to girls  Control: School smoking prevention program only | Intervention: Teacher, mass media  Control: Teacher | 4 years  Number of Sessions: 4 | 6 years | 0.56 (0.33, 0.96) |

**Legend:** DARE=Drug Abuse Resistance Education; ESFA=European Smoking Prevention Framework Approach; HPS=Health Promoting Schools; NR=Not Reported; PASE= Substance Abuse Prevention in the School; RCT=Randomized Controlled Trial; RR=Risk Ratio; TNT= Towards No Tobacco
